# Physicochemical properties and antibiofilm activity of mineral trioxide aggregate associated with farnesol

**DOI:** 10.1590/1807-3107bor-2024.vol38.0066

**Published:** 2024-08-05

**Authors:** Gabriela Mariana CASTRO-NÚÑEZ, Mário TANOMARU-FILHO, Gisselle Moraima CHÁVEZ-ANDRADE, Fernanda Ferrari Esteves TORRES, Roberta BOSSO-MARTELO, Juliane Maria GUERREIRO-TANOMARU

**Affiliations:** (a) Universidad Catolica de Santa Maria – UCSM, School of Dentistry, Arequipa, Peru.; (b) Universidade Estadual Paulista – UNESP, School of Dentistry, Department of Restorative Dentistry, Araraquara, SP, Brazil.; (c) Universidade Federal da Bahia – UFBA, School of Dentistry, Department of Dentistry, Salvador, BA, Brazil.

**Keywords:** Biofilms, Dental Materials, Farnesol

## Abstract

This study assessed the physicochemical and antibiofilm properties of white mineral trioxide aggregate (MTA) associated with 1 or 2% of farnesol. Setting time was evaluated based on ISO 6876/2012. Radiopacity was evaluated by radiographic analysis. pH was assessed after time intervals of 1, 3, 7, 14, 21, and 28 days. Solubility (% mass loss) and volumetric change (by micro-CT) of the cements were evaluated after immersion in distilled water. The presence of voids inside the materials was assessed by using micro-CT. Antibiofilm activity against *Enterococcus faecalis* was evaluated by crystal violet assay and the modified direct contact test performed with biofilm previously formed on bovine root dentin for 14 days. Data were submitted to ANOVA/Tukey tests with 5% significance level. The incorporation of farnesol into MTA increased its setting time, but decreased its solubility at 30 days and its volumetric loss in all periods (p < 0.05). Radiopacity and solubility after 7 days were similar among the materials (p > 0.05). The association of farnesol showed the highest pH value after 1 and 3 days (p < 0.05). The association of farnesol with MTA promoted a decrease in the presence of voids, and increased the antimicrobial activity on biofilm biomass of *E. faecalis* (p < 0.05). In conclusion, the addition of farnesol can be suggested to improve the antimicrobial properties and the consistency of MTA.

## Introduction

Calcium silicate–based endodontic materials have many clinical applications for endodontic procedures.^1^ Therefore, these materials must have adequate physicochemical properties, such as setting time, solubility and radiopacity.^2^ Fast setting after insertion of the endodontic cements is desirable since a long setting time can result in a higher solubility rate.^3^ High solubility can negatively affect the stability of the material.^4^ In addition, ideally, these materials should have sufficient(?) radiopacity to allow their visualization after clinical application^5^ and follow-up evaluations.^2^ MTA is a calcium silicate-based endodontic reparative material that has biocompatibility^6,7^ but poor handling characteristics.^3^ Moreover, although the methods used to evaluate the antimicrobial properties of MTA are limited, the material has demonstrated limited antimicrobial activity.^8^ Therefore, strategies are needed to improve its antimicrobial properties since it is mainly applied in areas that present microorganisms in the form of biofilm.^8^

Some associations have been proposed to improve consistency of MTA.^3^ Moreover, different substances have been assessed with the purpose of increasing its antimicrobial activity.^9^ Chlorhexidine and silver nanoparticles may improve antimicrobial effectiveness of MTA against microorganisms present in endodontic infections.^10,11^ Although the addition of 2% chlorhexidine increased its activity against *E. faecalis*,^12^ this association was shown to lead to cytotoxicity^13^ and interfered in the physical properties of MTA.^14^ Natural extracts of Casearia sylvestris added to MTA increased the antimicrobial effect. However, some changes on physicochemical properties were observed, such as an increase in the setting time, and the need for a specific protocol to obtain this extract.^6^

Bacterial resistance to antibiotics has motivated the evaluation of new antimicrobial agents, such as Essential Oils, which are natural products that protect plants against diseases. Essential oils have been used for therapeutic and medicinal purposes.^15^ Farnesol is a natural sesquiterpene alcohol found in essential oils of citric fruits, which has shown antitumoral, antimicrobial and antibiofilm activities.^15^ Farnesol acts on the cell membranes of bacteria, compromising their integrity and leading to release of the intracellular content.^16^ Farnesol affects the structure of biofilm, reducing biomass, killing bacteria in biofilms, without reducing their susceptibility over the course of cellular generations.^16^

Farnesol has been shown to have low cytotoxicity and antimicrobial activity, allowing its use during irrigation of the root canals.^17,18^ Although it is an active ingredient with promising properties, there is no study that has evaluated the effect of associating farnesol with calcium silicate-based reparative materials on their physicochemical properties and antibiofilm activity. Therefore, the aim of this study was to evaluate the physicochemical properties and the antibiofilm activity against *E. faecalis* of MTA associated with farnesol (1 and 2%). The experimental hypothesis was that the association of farnesol with MTA would not change the physicochemical properties but would increase the antibiofilm activity of MTA.

## Methods

The Material manufacturers and their proportions are described in Table 1. The powder/liquid proportion for MTA was used according to the manufacturer’s recommendation. To determine the powder/liquid proportion of the materials associated with farnesol, a test was conducted. Different proportions were used to obtain a consistency considered satisfactory for clinical application in retrograde filling, according to the results observed in the associations. Farnesol solution was prepared according to previous studies.^17,18^

**Table 1:** Endodontic materials, their manufacturers and proportion used.

Material	Manufacturers	Proportion
White MTA (MTA)	Angelus, Londrina, Brazil	1 g powder:
		330 µL distilled water
White MTA + Farnesol 1%	MTA: Angelus, Londrina, Brazil	1 g powder:
	Farnesol: Sigma-Aldrich, St. Louis, USA	330 µL distilled water:
		15 µL farnesol
White MTA + Farnesol 2%	MTA: Angelus, Londrina, Brazil	1 g powder/
	Farnesol: Sigma-Aldrich, St. Louis, USA	330 µL distilled water/
		30 µL farnesol

### Evaluation of physicochemical properties

#### Setting time

To determine the setting time, metal rings 10 mm in internal diameter by 1 mm high were used (n = 6). Setting time was evaluated based on the ISO 6876/2012 specification.^19^ To evaluate the initial setting time, a Gilmore needle with mass of 100 ± 0.5 g and tip diameter of 2 ± 0.1 mm was supported on the cement surface. To assess the final setting time, the same procedure was repeated, using a Gilmore needle with mass of 456 ± 0.5g and tip diameter of 1 ± 0.1 mm. The materials were kept in an oven at 37°C ± and 95% ± 5% humidity, during analysis and the needle was cleaned between the analyses. The setting time was determined by starting from beginning of manipulating the cements up to the time when the marks of the needles could no longer be observed on the cement surface.

#### Solubility

The solubility test was performed using specimens measuring 7.75 mm in diameter and 1.5 mm high^20^ that had an impermeable nylon thread included in the middle of the material (n = 6). The test was performed based on Zordan-Bronzel et al.^21^ The specimens were stored in an oven (37 ± 1ºC and relative humidity),for a period three times longer than the duration of their setting time. The specimens were placed in a desiccator containing silica gel under vacuum. They were weighed on a precision balance (Adventurer AR2140, Ohaus Corporation, Parsippany, USA), until stabilization of the initial mass (approximately 72 hours). Afterwards the samples were suspended in 7.5 mL of distilled and deionized water (37 ºC), without contact between the material and internal surface of the receptacle, for time intervals of 7 and 30 days.^22^ After each time interval, the specimens were washed with distilled water, dried with absorbent paper. They were placed in the desiccator again, until final stability of the mass was obtained (approximately 72 hours). The loss of mass was expressed in percent of original mass.

## Volumetric change and presence of voids: micro-CT analysis

Epoxy resin blocks were fabricated with standard cavities measuring 1 mm in diameter and 3 mm deep, simulating retrograde cavities.^22^ A single operator, who was previously trained and calibrated, filled the cavities with each material using a condenser kit (Ref.: 324501, numbers 2, 3 and 4; Golgran; São Caetano do Sul, Brazil). The samples were kept in an oven at 37º C and relative humidity for a period three times longer than the duration of their setting time.

The specimens (n = 6 ) were scanned with a microcomputed tomography system (micro-CT. SkyScan 1176. Bruker, Kontich, Belgium). The scanning procedure was performed using 50 kV X-ray tube voltages and 500 mA anode current; aluminum filter of 0.5; isotropic voxel of 18 mm; and a 360º evolution cycle. After the first scanning, the samples were placed in closed plastic flasks containing 7.5 mL of distilled water, and kept in an oven at 37°C for 7 and 30 days. After these periods, the samples were scanned again.

**Figure 1:**
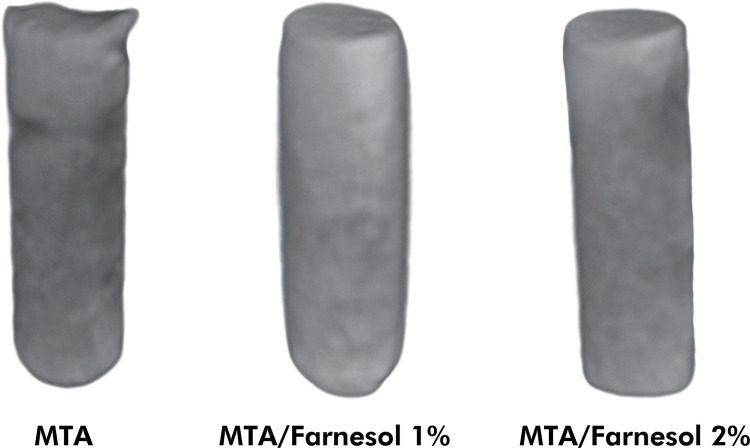
Illustration of tridimensional micro–computed tomography images of samples of cavities filled with MTA, MTA + Farnesol 1% and MTA + Farnesol 2%.

**Figure 2:**
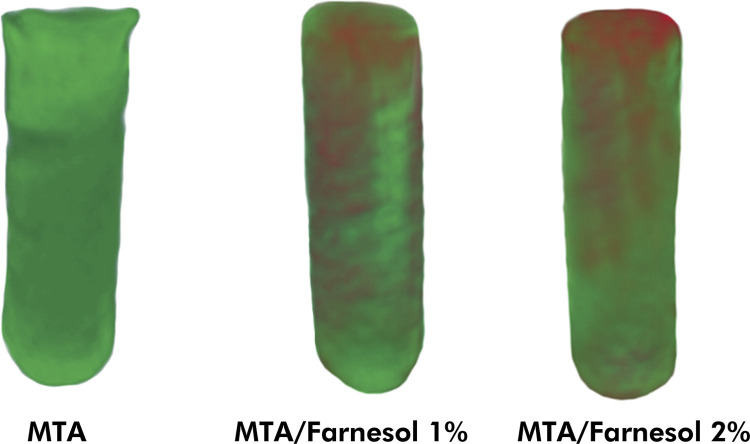
3D models using CTVox software, illustrating MTA, MTA + Farnesol 1% and MTA + Farnesol 2%, before (green) and after (red) immersion in distilled water for 30 days during volumetric change evaluation.

The images obtained were reconstructed with NRecon software (V1.6.4.7; Bruker). The correction parameters for smoothing, beam hardening, and ring artifacts were defined for each material. The same parameters were used for the same material in the different periods. The reconstructed images were superposed at the different periods by using the Data Viewer program (V1.5.2.4; Bruker). The images were used for quantitative analysis of the samples and allowed the total volume (mm^3^) and the presence of voids (%) in the materials to be calculated by using the CTAn software (V1.11.8; Bruker). The CTVox software (V3.2.0; Bruker) was used to create three-dimensional models of the cavities after filling (Figure 1). The presence of voids (%) was calculated considering the baseline, and the volumetric change between the baseline and the experimental time intervals is represented in Figure 2.

## Radiopacity

Six specimens measuring 10 mm in diameter and 1 mm high were manufactured and kept in an oven at 37ºC ± 1ºC and relative humidity, for a period of time three times longer than the duration of their setting time.^21^ A test specimen of each material together with an aluminum scale with variations in thickness from 2 to 16 mmAl, were radiographed on an occlusal film (Insight – Kodak Comp, Rochester, USA). An X-ray unit (Instrumentarium Dental, Tuusula, Finland) operating at 60 kV, 7 mA, 0.32 pulses per second, and a focus-film distance of 33 cm was used. The films were processed in a standard automatic processor (Dent-X 9000, Dent-X, Elmsford, USA). The radiographs were digitized, and the images were imported into the Image Tool version 3.0 software (UTHSCSA, San Antonio, USA). The area of each degree of the aluminum scale and the area of the cements were selected to determine the radiopacity of the materials, expressed as equivalent thickness of aluminum (in mm).

## pH

For the pH test, polyethylene tubes 10 mm long and 1 mm in diameter were filled with each material (n = 10). Each tube was immersed in 10 mL of distilled and deionized water, of which the pH was previously checked. Then the tubes were kept in an oven at 37 ºC throughout the experimental periods. After each time interval (1, 3, 7, 14, 21 and 28 days), the polyethylene tubes were removed from the flasks and put into a new flask with 10 mL distilled and deionized water. Flasks containing only distilled and deionized water were used as control. pH was measured in triplicate with a digital pH meter (Digimed DM-21. Digicrom Analítica Ltda., São Paulo, Brazil) at an ambient temperature of 25°C. The test was performed based on Zordan-Bronzel et al.^21^

## Evaluation of antimicrobial properties

For antimicrobial evaluation, the strain of *E. faecalis* (ATCC 29212) was used. The bacteria were reactivated in sterile brain-heart infusion (BHI) (Difco Kansas City, MO, USA) until the logarithmic stage of growth was reached; when the inoculum was standardized for the experiments, at the concentration of 10^8^ colony forming unit (CFU) mL^-1^in a spectrophotometer (ELx800; Bio-Tek Instruments, Winooski, USA).

## Crystal violet assay

Discs (n = 8) of the materials measuring 5 mm in diameter and 1 mm high were fabricated,, and then kept in an oven at 37ºC and 100% humidity for 48 hours. Subsequently both sides of the discs were sterilized by means of ultraviolet radiation for 30 minutes.

The inoculum was distributed in 96-well culture plates (200 mL in each well), and the plates were incubated at 37ºC for 48 hours to allow biofilm formation on the bottom of the well. After this period, the wells were washed and the discs were placed in the wells in contact with the biofilm and 20 µL of saline solution at 37°C for 24 h.

To evaluate the residual biofilm, the wells were washed and stained with 0.1% crystal violet solution at ambient temperature for 20 minutes. After washing and drying, the dye on the adherent cells was solubilized with 200 µL of 33% acetic acid for 5 minutes. The biomass of the remaining biofilm was measured by absorbance (570 nm) in a spectrophotometer (Elx800; Bio-Tek Instruments, Winooski, USA). Saline solution was used for positive control.

## Modified direct contact test

Bovine maxillary incisors were used to obtain radicular dentin blocks (n=6) measuring 5 mm x 5 mm x 0.7 mm (width x length x thickness); the blocks were immersed in 2.5% NaOCl (AraQuímica, Araraquara, Brazil) and 17% EDTA (Biodinâmica, Ibiporã, Brazil) for 5 minutes and then abundantly washed with distilled water. Finally, they were distributed into test tubes and sterilized by autoclave at 121ºC.

Discs measuring 7 mm in diameter and 1 mm thick were fabricated of the materials, kept in an oven at 37ºC ± 1 °C for 48 hours; and sterilized by means of ultraviolet radiation on each side for 30 minutes.

In a 24-well cell culture plate, 2,0 mL of the inoculum was dispensed, leaving the dentin blocks completely submersed. The plates were incubated with orbital agitation (incubator model Q816M20, Quimis Aparelhos Científicos Ltda., Diadema, SP, Brazil) in a microaerophilic environment at 37ºC for 14 days. The culture medium was renewed every 48 hours, without the addition of bacteria.

On day 14 the dentin blocks were washed and the cement discs were placed on the dentin with 20 μL of sterile saline solution. The time of contact was 15 hours and the plates were kept in an oven at 37°C. For the control group Teflon discs of the same weight and size were used.

After the contact period had elapsed, each dentin block was placed in a test tube with 1 mL sterile saline solution and glass pearls. The tubes were agitated in a vortex (model Q220, Quimis Aparelhos Científicos Ltda., Diadema, Brazil) for 1 minute. After this, serial decimal dilution was performed; three aliquots of 20 μL of each dilution were distributed on Petri dishes containing the culture medium Tryptic Soy Ágar (Difco Kansas City, USA). The plates were incubated at 37 ºC for 48 hours.

Of the number of colonies that formed in the dilution, between 5 and 50 colonies were quantified. From the mean value quantified, the number of CFU mL^-1^were calculated. The data were submitted to logarithmic transformation and the result of each group was calculated from the mean value of its four specimens.

## Statistical analysis

The results obtained were submitted to a normality test, and then to the two-way ANOVA statistical test and Tukey multiple comparison test, with 5% significance level.

## Results

### Physicochemical properties

The results of physicochemical properties are described in Table 2 and Table 3.

**Table 2:** Means and standard deviation of setting time, solubility, volumetric change, presence of voids, and radiopacity observed in the different cements.

Tests/materials	MTA	MTA + Farnesol 1%	MTA + Farnesol 2%
Initial setting time (min)	16.17 ± 1.17^a^	25.67 ± 2.42^b^	26.33 ± 2.94^b^
Final setting time (min)	276.20 ± 53.71^a^	480.00 ± 34.99^b^	441.70 ± 29.94^b^
Solubility (% mass loss) 7 days	-1.53 ± 0.35^aA^	-1.46 ± 0.45^aA^	-1.35 ±0.48^aA^
Solubility (% mass loss) 30 days	3.87 ± 1.10^aB^	1.37 ± 0.32^bB^	-0.91 ± 0.36^cA^
Volumetric change 7 days (%)	-0.49 ± 1.03^bA^	0.55 ± 0.69^aA^	0.73 ± 0.72^aA^
Volumetric change 30 days (%)	-0.20 ± 1.58^aA^	1.35 ± 1.48^bA^	2.53 ± 1.05^bB^
Voids (%)	5.61 ± 2.25^a^	0.31 ± 0.18^b^	0.50 ± 0.40^b^
Radiopacity (mm Al)	5.09 ±0.17^a^	5.43 ± 0.35^a^	5.25 ± 0.27^a^

Different capital letters in the same column indicate statistically significant difference among the periods (p < 0.05). Different lower-case letters on the same row indicate statistically significant difference among the cements (p < 0.05). Negative values in the volumetric change test indicate volume loss.

**Table 3:** Means and standard deviation of pH values observed at the different experimental periods.

Periods	MTA	MTA + Farnesol 1%	MTA + Farnesol 2%	Control
1 day	10.72 ± 0.09^bA^	10.92 ± 0.08^aA^	10.90 ± 0.16^aA^	6.71 ± 0.24^cA^
3 days	10.72 ± 0.11^bA^	10.93 ± 0.10^aA^	10.93 ± 0.17^aA^	6.75 ± 0.17^cA^
7 days	9.97 ± 0.20^aB^	10.04 ± 0.21^aB^	9.90 ± 0.20^aB^	6.87 ± 0.07^bA^
14 days	9.90 ± 0.07^aB^	9.91 ± 0.10^aB^	9.90 ± 0.13^aB^	6.80 ± 0.24^bA^
21 days	9.05 ± 0.16^aC^	8.99 ± 0.21^aC^	9.04 ± 0.13^aC^	6.82 ± 0.08^bA^
28 days	8.95 ± 0.16^aC^	8.80 ± 0.12^aD^	8.94 ± 0.22^aC^	6.73 ± 0.06^bA^

Different capital letters in the same column indicate statistically significant difference among the periods (p < 0.05). Different lower-case letters on the same row indicate statistically significant difference among the cements (p < 0.05).

### Setting time

MTA had the shortest setting time (p < 0.05). MTA + Farnesol 1% and MTA + Farnesol 2% were similar (p > 0.05).

### Solubility

Relative to solubility, at 7 days the materials were similar (p > 0.05), showing an increase in mass. After 30 days, MTA and MTA + Farnesol 1% had mass loss, with the highest values for MTA (p < 0.05). MTA + Farnesol 2% had an increase in mass (p < 0.05) and maintained its values in the time interval between 7 and 30 days of immersion (p > 0.05).

### Volumetric change and presence of voids: micro-CT analysis

MTA showed volume loss, while MTA + Farnesol 1% and MTA + Farnesol 2% had an increase in volume (p > 0.05), with higher values than MTA (p < 0.05). When the time intervals were compared, MTA had values below 0.5% at 7 and 30 days (p > 0.05). MTA + Farnesol 1% maintained its values between 7 and 30 days (p > 0.05), while MTA + Farnesol 2% had a higher level of increase in volume after 30 days (p < 0.05). Relative to the percentage of voids, a decrease was observed in the association with farnesol at 1 and 2% (p > 0.05) in comparison with MTA (p < 0.05).

### Radiopacity

The three materials were similar and presented radiopacity exceeding 5 mm Al (p > 0.05).

### pH

During all the experimental time intervals, the control group (distilled water) had the lowest pH values (p < 0.05). In the time intervals of 1 and 3 days, MTA + Farnesol 1% and MTA + Farnesol 2% had the highest alkalinization capacity (p > 0.05), in comparison with MTA (p < 0.05). At 7, 14, 21 and 28 days, all the materials were similar (p > 0.05).

### Antimicrobial properties

#### Crystal violet assay and modified direct contact test

The addition of farnesol in both concentrations showed similar results (p > 0.05), by increasing the capacity for reducing the biomass of *E. faecalis*, and significantly reducing the CFU mL^-1^when compared with MTA (p < 0.05), which also showed significant difference when compared with the control (p < 0.05) (Table 4).

**Table 4:** Means and standard deviation of crystal violet assay (Absorbance), and Modified Direct Contact Test (CFU mL-1 log10).

Test	MTA	MTA + Farnesol 1%	MTA + Farnesol 2%	Control
CV	0.299 ± 0.05^b^	0.150 ± 0.04^a^	0.152 ± 0.02^a^	0.462 ± 0.03^c^
MDCT	5.685 ± 0.16^b^	4.834 ± 0.37^a^	4.617 ± 0.33^a^	7.806 ± 0.39^c^

Different letters on the same row indicate statistically significant difference between the cements (p < 0.05).

## Discussion

The experimental hypothesis was partially accepted since the association of farnesol with MTA changed some physicochemical properties and increased its antibiofilm activity. This association improved the physicochemical and antimicrobial properties of MTA. Biological properties of MTA in association with farnesol was not evaluated in the present study. However, farnesol presented low cytotoxicity without genotoxic effect on fibroblast-like cells.^17^

Initial and final setting time values were higher for the associations of MTA with farnesol. In a similar manner, the addition of propylene glycol to MTA increased the setting time.^3^ Thus, the incorporation of this sesquiterpene alcohol could promote a longer hydration process, thereby increasing the setting time since MTA releases calcium hydroxide, which may have no conductivity in alcohols.^3^

The main disadvantage relative to the longer setting time is the increase in solubility.^3^ However, a decrease in the mass loss was observed at 30 days after the incorporation of farnesol at 1% and at 2%. This finding could be related to the fact that oils are not water-miscible, thus they increase the washout resistance of the cements.^23^ The solubility values of MTA corroborate the findings of a previous study.^22^ Nevertheless, the solubility test that considers the mass loss after immersion in distilled water presents some limitation, since calcium silicate-based materials can absorb water, which affects the final mass.^5^ The contact area between cement and water during the test can be considered another disadvantage,^22^ since it does not represent the clinical condition.^4^ Therefore, in our study, we used micro-CT as a complementary 3D method to evaluate the volumetric change of the materials,

During the volumetric change assessment, the cements were evaluated inside simulated root-end cavities, thus simulating clinical condition.^5,22^ Our results showed that the association of farnesol promoted an increase in volume, in agreement with the findings of the solubility test. These results suggested an expansion of MTA after the addition of farnesol, which could improve the marginal adaptation and sealing ability of this material.^24^ In the two time intervals evaluated in the present study, the materials presented a volumetric change of less than 3%. Previous studies also observed volume loss below 3% for MTA”.^5,6,22^ Moreover, the addition of farnesol in the two concentrations evaluated improved the handling characteristic of MTA. Duarte et al.^3^ observed that propylene glycol improved the handling characteristics of MTA, probably due to finer particles obtained after the association. This improved consistency favored the filling ability of MTA, as observed in the lower percentage of voids observed in the micro-CT evaluation.

The radiopacity of the materials was also evaluated since it is important to be able to distinguish an endodontic material from the surrounding structures.^25^ The results of the present study showed that all the materials were similar, showing that the addition of farnesol did not interfere in this property. These results exceeded the minimum value of 3 mm Al recommended by the ISO 6876 specification. In studies that evaluated the radiopacity of MTA in blood and distilled water, values higher than 3 mm Al were observed.^25^

The pH test was conducted in accordance with a previous study.^26^ Our results showed that the addition of farnesol increased the alkalinization of the medium in the time intervals of 1 and 3 days, which could possibly be related to the longer setting time of the materials.^3^ These higher pH values could improve the antimicrobial activity. However, in the other time intervals evaluated, the alkalinization ability among the materials was similar.

The crystal violet and modified direct contact tests are methods used to evaluate the antibiofilm activity of different substances in the biomass of biofilm.^15,18^ The antibiofilm activity against the biomass of *E. faecalis* is used to evaluate antimicrobial activity of endodontic materials.^9^ The addition of farnesol to MTA showed an increase in its antimicrobial activity. Farnesol evaluated as irrigating solution also showed antimicrobial and antibiofilm effects.^18^

All the materials evaluated had the capacity to reduce the biomass of biofilm. However, the association of farnesol at 1% and 2% had better activity when compared with MTA. The modified direct contact test had similar results, indicating that the incorporation of farnesol in the two concentrations decreased the CFU mL^-1^when in contact with the biofilm of *E. faecalis*. This results could be explained since farnesol plays a role in biofilm virulence, formation and competence,^27^ by affecting the cell viability and the biomass of the biofilm, preventing the formation of biofilms, and by acting on previously formed biofilms. Farnesol reduced the production of biofilm matrix which can accumulate in the bacterial cell membrane, causing the loss of intracellular content.^13,16^ Furthermore, farnesol blocked efflux pumps, downregulated biofilm- and efflux pump- associated genes.^27^

In vitro studies should not be directly extrapolated to clinical situations. However, these data serve as a reference for future investigations. The findings of this laboratory research must be complemented with further studies assessing the effect of farnesol on the biological properties of calcium silicate-based materials, and subsequent in vivo investigations in order to confirm its clinical application.

## Conclusions

Within the limitations of this in vitro study, it was concluded that the addition of farnesol increased MTA antimicrobial activity and improved its consistency. The alternative use of this essential oil changed some physicochemical properties of MTA, therefore, complementary studies are required for future clinical applications, and for contributing to the long-term prognosis of endodontic treatment.
